# Comprehensive metabolomic and lipidomic alterations in response to heat stress during seed germination and seedling growth of Arabidopsis

**DOI:** 10.3389/fpls.2023.1132881

**Published:** 2023-03-29

**Authors:** Wenjuan Qian, Yuxuan Zhu, Qinsheng Chen, Shuaiyao Wang, Longlong Chen, Ting Liu, Huiru Tang, Hongyan Yao

**Affiliations:** ^1^ State Key Laboratory of Genetic Engineering, School of Life Sciences, Human Phenome Institute, Metabonomics and Systems Biology Laboratory at Shanghai International Centre for Molecular Phenomics, Zhongshan Hospital, Fudan University, Shanghai, China; ^2^ SCIEX, Analytical Instrument Trading Co., Ltd, Shanghai, China

**Keywords:** seed germination, seedling growth, heat stress, arabidopsis, phenotyping, metabolite, lipidome

## Abstract

Temperature affects seed germination and seedling growth, which is a critical and complex stage in plant life cycle. However, comprehensive metabolic basis on temperature implicating seed germination and seedling growth remains less known. Here, we applied the high-throughput untargeted metabolomic and advanced shotgun lipidomic approaches to profile the Arabidopsis 182 metabolites and 149 lipids under moderate (22°C, 28°C) and extreme high (34°C, 40°C) temperatures. Our results showed that a typical feature of the metabolism related to organic acids/derivates and amines was obviously enriched at the moderate temperature, which was implicated in many cellular responses towards tricarboxylic acid cycle (TCA), carbohydrates and amino acids metabolism, peptide biosynthesis, phenylpropanoid biosynthesis and indole 3-acetate (IAA) biosynthetic pathway. Whereas, under extreme high temperatures, there was no seed germination, but 148 out of total 182 metabolites were highly enriched, involving in the galactose metabolism, fatty acid degradation, tryptophan/phenylalanine metabolism, and shikimic acid-mediated pathways especially including alkaloids metabolism and glucosinolate/flavone/flavonol biosynthesis. Phosphatidylcholine (PC) and phosphatidylethanolamine (PE) also exhibited the gradually increased tendency from moderate temperatures to extreme high temperatures; whereas phosphatidylserine (PS), phosphatidic acid (PA), phosphatidylglycerol (PG), monogalactosyldiacylglycerol (MGDG), digalactosyldiacylglycerol (DGDG) and sulfoquinovosyldiacylglycerol (SQDG) were contrary to decrease. Another typical feature of the distinguished metabolites between 22°C and 28°C, the TCA, disaccharides, nucleotides, polypeptides, SQDG and the biosynthesis of fatty acids and glucobrassicin-mediated IAA were obviously decreased at 28°C, while amino acids, trisaccharides, PE, PC, PA, PS, MGDG, DGDG and diacylglycerol (DAG) preferred to enrich at 28°C, which characterized the alteration of metabolites and lipids during fast seedling growth. Taking together, our results provided the comprehensive metabolites phenotyping, revealed the characteristics of metabolites necessary for seed germination and/or seedling growth under different temperatures, and provided insights into the different metabolic regulation of metabolites and lipid homeostasis for seed germination and seedling growth.

## 1 Introduction

Climate projections for Europe predict temperature increases (Pachauri et al., 2016), and extreme heat not only dramatically reduces plant growth, but also has a strong impact on the most vulnerable biological processes of plant life cycle, such as the initial critical stages, seed germination and seedling growth (Dreesen et al., 2014).

Seed germination and seedling growth are complex biochemical and physiological processes generally involving many endogenous transformations of plant metabolites and metabolism pathways, such as the differential hydrolysis of starch, lipids and proteins (Zinta et al., 2018). Several endogenous plant metabolites, especially hormones, have been discovered to involve in seed germination. The alteration in abscisic acid (ABA) biosynthesis and/or metabolism pathway could greatly influence seed germination (Holdsworth et al., 2008), such as, the Arabidopsis mutations of ABA biosynthetic genes *ZEP*/*ABA1* (Koornneef et al., 1982) and *NCEDs* (Frey et al., 2012) promoting germination, whereas over-expression of ABA biosynthetic genes delayed seed germination (Martínez-Andújar et al., 2011). Genetic and physiological studies have shown that ethylene and brassinosteroid affected the balance of ABA/gibberellins (GAs) through counteracting ABA effects to promote germination (Koornneef et al., 2002). Loss of function in GAs biosynthetic genes such as ent-copalyldiphosphate synthase (CPS/GA1), ent-kaurene synthase (KS/GA2), ent-kaurene oxidase (KO/GA3) and gibberellin-3-oxidases (GA3ox1 and GA3ox2) inhibited seed germination (Koornneef and van der Veen, 1980; Yamauchi et al., 2004). Ethylene and its immediate precursor (1-aminocyclopropane-1-carboxylic acid, ACC) completely improved seed germination (Arc et al., 2013). The ethylene mutants affected ethylene metabolism or signaling and also showed the sensitivity to ABA (Corbineau et al., 2014).

Seed germination and seedling growth were largely affected by external factors, and germination thermoinhibition process was associated with temperature-sensitive expression of ABA, GA, and ethylene biosynthesis, metabolism, and response genes in Lettuce (Argyris et al., 2008). The thermoinhibition process was alleviated by application of ABA biosynthesis inhibitor fluridone, exogenous GAs or ethylene (ACC) (Gonai et al., 2004; Kpczyska et al., 2006; Toh et al., 2008). Further studies showed that appropriate temperature-induced dormancy release and germination mostly might depend on the re-localization of ABI5 (ABA-INSENSITIVE 5) and DELLA protein RGL2 (RGL2) in the cytoplasm allowing sensitivity change to ABA and GAs, and the localization of ACC oxidase (ACO) in the meristematic zone to allow on-site ethylene production in sunflower (Xia et al., 2019). However, an increase in excess temperature reduced seed germination through promoting the accumulation of ABA and reactive oxygen species (ROS) in rice (Liu et al., 2019). Except ABA, the contents of superoxide anion ( ${\text{O}}_{2}^{\mathrm{\cdot -}}$ ), hydrogen peroxide (H_2_O_2_) and malondialdehyde were increased in germinating seeds under high temperature (Liu et al., 2019). Temperature and hormone signaling including auxin, Brassinosteroids (BR) and GA interconnected to act the regulation of plant thermomorphogenesis during seedling establishment (Stavang et al., 2009), and BRs regulated temperature-induced hypocotyl elongation *via* BRASSINAZOLE RESISTANT 1 (BZR1) (Ibañez et al., 2018). As the metabolites, such as phytohormones, superoxides and malondialdehyde, were known to play crucial roles in seedling establishment, the characteristic phenotyping of metabolites would provide comprehensive metabolic basis onto thermotolerance during this process.

Lipids constitute membrane structure and also mediate multiple cellular signaling transduction. In seed germination and seedling growth, PA showed the lowest levels in imbibition and followed an increasing trend during seedling growth, while PE molecules showed the highest levels in imbibition and a general decreasing trend at later stages (Wang et al., 2016). Heat stress modulated the lipid composition to make membrane lipids less unsaturated and more oxidized acyl chains in Arabidopsis mature leaf (Shiva et al., 2020). Efficient enzymatic and non-enzymatic antioxidant defense systems played a crucial role in scavenging and detoxifying ROS to reduce membrane lipid peroxidation and maintain ROS homeostasis and redox signaling under abiotic oxidative stress (Hasanuzzaman et al., 2012). Lipids interconnected other signaling molecules to maintain metabolic homeostasis in plant development; whereas, whether and how lipids response to temperature during seed germination and seedling growth remains unknown.

Metabolomic and lipidomic analysis have become a powerful technology to comprehensively clarify plant dynamic development (Wang et al., 2016), plant reproductive development (Du et al., 2022), and pathogen and plant interaction (Liu et al., 2017). The electrospray ionization (ESI) coupled tandem mass spectrometry (MS/MS) features the potentially most sensitive, discriminating and direct method and widely used for the qualitative and quantitative analysis of subpicomole amounts of lipids with simple sample preparation (Kerwin et al., 1994; Welti and Wang, 2004).

In this study, we investigated the comprehensive metabolic and lipidomic profiles during seed germination and seedling growth through the systematic biology approaches of both high-throughput untargeted metabolomics and advanced ESI-based lipidomics. We demonstrated the characteristics of metabolites and lipids in response to moderate (22°C and 28°C) and extreme high (34°C and 40°C) temperatures during the both stages of seedling establishment, and the distinguished characteristics between 22°C and 28°C during seedling growth. Our findings provided a molecular characteristic phenotype for seedling establishment under heat stress, revealed the metabolic pathways of TCA, carbohydrates metabolism, peptide biosynthesis, and lipid homeostasis were crucially implicated in seed germination and seedling growth under heat stress, providing cues to further study functional mechanism of these cellular metabolic pathways.

## 2 Materials and methods

### 2.1 Plant materials and seed germination

Seed germination was carried out according to the previous study (Boyes et al., 2001). Briefly, seeds of *Arabidopsis thaliana* ecotype Columbia (Col) were surface-sterilized and kept in the dark at 4°C for 3 days. The seeds were then sown onto filter papers with pre-soaked sterile water, and placed at 22 °C under light for 3 hours before transferring to the dark chamber for another 21 hours at 22°C, 28°C, 34°C and 40°C, respectively.

### 2.2 Sample preparation

Total metabolites and lipids from 50 mg samples with whole tissue were extracted with methyl tert-butyl ether (MTBE) and methanol (MeOH). Briefly, 50 mg plant materials were mixed with 225 μL of ice-cold MeOH and homogenized for three times. The homogenate was then sonicated in an ice bath for 10 min. Added 750 μL pre-cooled MTBE and vortexed the mixture for 30 s and then kept on ice for 1 hour. The samples were vortexed for 30 s after adding 188 μL deionized water, and centrifuged at 14000 g for 10 min at 4°C. All experimental steps were performed on ice. The upper organic phase (800 μL) was carefully transferred to new tubes. The lower phase (200 μL) was transferred to additional new tubes. Repeat the above extraction steps twice. Finally, the three extractions were combined and dried with nitrogen gas.

The upper phase dried extract was dissolved in chloroform: methanol: 300 mmol/L ammonium acetate (300: 665: 35; v: v: v) for lipids analysis. The lower phase dried extract was dissolved in methanol: water (1:1) for metabolites analysis.

### 2.3 UPLC-QTOF/MS-based metabolomic analysis

The Shimadzu Nexera UHPLC system equipped with the 6600 QTOF mass spectrometer (AB Sciex) was applied for untargeted metabolomics analysis. 1 uL aliquots of sample was injected into a Waters Acquity UPLC T3 column (100mm×2.1mm, 1.7 μm) maintained at 45°C by gradient elution. Mobile phase A was deionized water with 5 mM ammonium acetate (NH_4_AC) and 0.02% formic acid, and phase B was acetonitrile: water (95:5, v/v) with 5 mM NH_4_AC and 0.02% formic acid. The flow rate was 0.3 mL/min. The ion source parameters were set as follows: scan range, 50-1200 Da; source temperature, 300°C; curtain gas, 35; collision gas, medium; two ion source gases, 60; ion spray voltage, 5500/-4500V; DP, 60; CE, 35 ± 15.

### 2.4 ESI/MS-based shotgun lipidomic analysis

The shotgun lipidomic analysis was carried out according to the previous study (Wang et al., 2016). Briefly, Shimadzu CBM-20A lite HPLC system coupled with a 6500 QTrap triple quadruple mass spectrometer was used to detect lipids. The mobile phase was methanol: dichloromethane (1:1, v/v) with 10 mM NH_4_AC. 50 μL aliquots of sample solution was injected and the flow gradient was performed with 7 μL/min for 6 min, and then 30 μL/min for 2 min and 7 μL/min for 1 min. MS/MS was triggered by an inclusion list encompassing corresponding MS mass ranges scanned in 1 Da increments. Both MS and MS/MS data were combined to monitor PA, PG, PI, MAG, DAG, TAG, MGDG and DGDG ions as ammonium adducts; PC, PE, PS and Cer as protonated ions; SQDG as deprotonated anions (**Supplemental Table S1**). The ion source parameters were set as follows: source temperature, 200°C; curtain gas, 20; collision gas, medium; two ion source gases, 15/20; ion spray voltage, 5500V; entrance potential, 10V.

### 2.5 Data progressing

The raw data of metabolomics was preprocessed by MetDNA2 (http://metdna.zhulab.cn/), identified according to the database, and some of them were confirmed by ZenoTOF 7600 (AB Sciex). The relative amount was comparatively quantified according to the peak intensity. The data of shotgun lipidomics was processed by LipidView Software (AB Sciex) for identification and quantification.

The data matrix of identified metabolites and lipids was further analyzed by univariate and multivariate statistical methods through website https://cloud.oebiotech.cn/ and R. Significantly altered variables were defined by the following criteria: *P* value < 0.05, FDR < 0.05 and Fold change > 1.5.

## 3 Results

### 3.1 Identification of metabolites and lipids during seed germination/seedling growth at four temperature conditions

**Figure 1:**
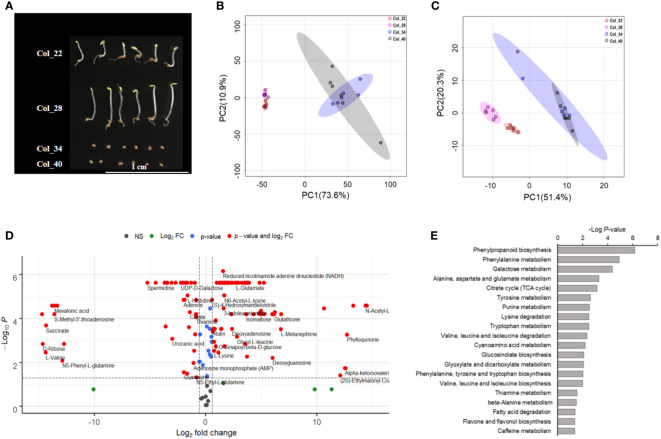
Global analysis of metabolite and lipids for Arabidopsis seeds germination/seedling growth under four temperatures conditions. **(A)** The observation of seeds germinated at 22°C, 28°C, 34°C and 40°C, respectively. Col_22 means seeds germinated at 22°C, Col_28 means seeds germinated at 28°C, Col_34 means seeds germinated at 34°C, Col_40 means seeds germinated at 40°C. **(B)** Principal component analysis of the inter-group metabolic differences during Arabidopsis seeds germination/seedling growth under four temperatures conditions. **(C)** Principal component analysis of the inter-group lipids differences during Arabidopsis seeds germination/seedling growth under four temperatures conditions. **(D)** Significantly distinct metabolites between moderate (22°C, 28°C), and extreme high temperatures (34°C, 40°C) were analyzed by volcano plot. X-axis was Log2 fold change and the y-axis was –Log10 *P* value. The red dots on the left indicated that metabolites of seeds germination at moderate temperature significantly reduced level, compared with the extreme high temperature. The red dots on the right indicated metabolites level increased significantly. Blue dots represented metabolites with only *P*<0.05 and green dots represent metabolites with only FC>1.5. Gray dots referred to all other identified metabolites with no significant changes between moderate and extreme high temperatures. **(E)** Analysis of metabolic pathways based on the differences of metabolites between moderate (22°C, 28°C) and extreme high temperatures (34°C, 40°C). Bioinformatic analysis was performed using the MetDNA tools at http://metdna.zhulab.cn/.

Seeds were germinated at 22°C, 28°C, 34°C and 40°C, respectively. As shown in **Figure 1A**, both 22°C and 28°C were suitable for seed germination, while the seeds were not germinated at either 34°C or 40°C. The hypocotyl of seedlings was longer at 28 °C than that at 22°C. Total 182 metabolic molecules were identified by using high-throughput untargeted metabolomic approach, which covered the metabolism of amino acids, amines, peptides, carbohydrates, lipids, nucleosides, organic acids, indoles, benzene/derivatives and flavones (**Supplementary Data 1**).

For shotgun analysis, we innovatively introduced specific ion pairs, and thus identified both glycolipids and glycerides in one injection, which further developed the previous approaches of ESI/MS for glycolipids and LC/MS for glycerides, respectively. Total 149 lipids including 13 PAs, 13 PCs, 18 PEs, 11 PGs, 2 PIs, 4 PSs, two subclasses of glycerides (22 DAGs, 25 TAGs), three subclasses of galactosyldiacylglycerides (17 MGDGs, 24 DGDGs, 1 SQDGs) and 2 ceramides (Cer 34:1, Cer 38:1) were identified (**Supplementary Data 2**).

### 3.2 Global analysis of metabolites and lipids during seed germination/seedling growth under four temperature conditions

Principal component analysis (PCA) was applied to analyze the metabolites which drove the group separation. PC1 and PC2 captured 73.6% and 10.9% of the total variance in the metabolites of FW-normalized dataset, respectively (**Figure 1B**). For lipids, PC1 and PC2 captured 51.4% and 20.3% of the total variance in the FW-normalized dataset, respectively (**Figure 1C**). The analysis showed that all samples were definitely divided into two clusters, grown at 22°C and 28°C (moderate temperature), and grown at 34°C and 40°C (extreme high temperature), respectively, which demonstrated that the metabolites and lipids were obviously distinct between moderate and extreme high temperatures.

The volcano plot further exhibited 148 metabolites significantly altered between moderate and extreme high temperatures (red points in **Figure 1D**, **Supplementary Data 3**). A total of 94 molecules dramatically increased in seeds grown at extreme high temperature, which included carbohydrates (D-Gal alpha 1->6D-Gal alpha 1->6D-Glucose, maltotriose, mannobiose, stachyose, raffinose), flavonoids (rutin, quercitrin, quercetin) and tryptophan metabolic derivatives (kynurenic acid, tryptamine) (**Supplementary Data 3**), whereas 54 metabolites significantly decreased at these extreme high temperatures. Specifically, the phenylpropanoid biosynthesis (sinapate, 4-hydroxycinnamic acid, spermidine), TCA (succinate, citrate, mevalonic acid) tended to have low levels at 34°C and 40°C (**Supplementary Data 3**). However, metabolites, such as amino acids, indoles, benzoic acids and their derivatives involving in different metabolic pathways changed variously (**Figure 1D**, **Supplementary Data 3**).

Based on the differential metabolites analyzed by volcano analysis, we further performed the pathway enrichment analysis to investigate the important roles of altered metabolites during seedling establishment under the moderate and extreme high temperatures (**Figure 1E**). Of all these significant pathways, phenylpropanoid biosynthesis and phenylalanine metabolism showed obvious differences. Galactose metabolism associated with sugar metabolism during seed germination was also obviously altered. Other energy metabolic pathways such as TCA, glucosinolate biosynthesis pathway, glyoxylate and dicarboxylate metabolism pathways were also obviously different between 22~28°C and 34~40°C (**Figure 1E**). Multiple amino acid metabolic pathways including alanine, aspartate and glutamate metabolism, tyrosine metabolism, lysine degradation, tryptophan metabolism, valine, leucine and isoleucine degradation/biosynthesis, phenylalanine, tyrosine and tryptophan biosynthesis and beta-alanine metabolism were altered between 22~28°C and 34~40°C (**Figure 1E**), which suggested that amino acid metabolism played vital roles in maintaining metabolic homeostasis during temperature stress. The distinguished alteration of fatty acid degradation pathway indicated that lipid metabolism significantly acted on seed germination and seedling growth (**Figure 1E**). The shikimic acid-mediated metabolic pathways including flavonoid/flavonol biosynthesis and terpenoids were also implicated in temperature responses (**Figure 1E**).

### 3.3 Significant differences of metabolites during seed germination/seedling growth at four temperature conditions

To further investigate the significance of metabolites at different temperatures, the hierarchical heatmap analysis Z score scaled the fold changes for metabolites. The 148 out of total 182 metabolites were enriched at extreme high temperature, compared with moderate temperature (**Supplementary Figures 1, 2**), which involved the metabolomic pathways of galactose metabolism (sucrose, raffinose, stachyose, D-Gal alpha 1->6D-Gal alpha 1->6D-Glucose), tryptophan metabolism (tryptophan, tryptamine, kynurenic acid, 3-indoleacetonitrile), vitamins metabolism (thiamin diphosphate, 2-alpha-hydroxyethy-thiamine diphosphate, thiamine), glucosinolate, and phenylalanine, tyrosine and tryptophan biosynthesis (methionine, tryptophan, phenylalanine), flavone and flavonol biosynthesis (quercetin, quercitrin, rutin), valine, leucine and isoleucine degradation (thiamin diphosphate, 2-Methylpropanoyl-CoA, 2-Methyl-1-hydroxybutyl-ThPP), lysine degradation (lysine, butanoyl-CoA, N6-Acetyl-L-lysine), and fatty acid degradation (butanoyl-CoA, hexanoyl-CoA) (**Supplementary Figures 1, 2**). These results demonstrated that many metabolic pathways, especially the pathways of secondary metabolites, were implicated in the response to extreme high temperature during seedling establishment.

**Figure 2:**
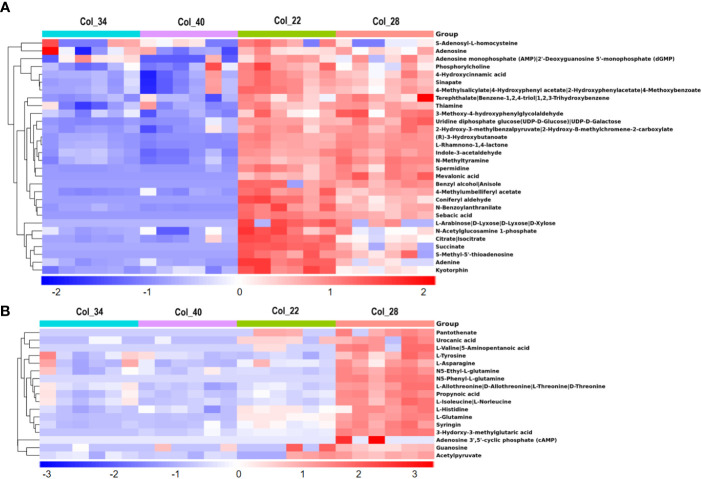
Comparative analysis of metabolites for Arabidopsis seeds germination/seedling growth between moderate and extreme high temperatures. **(A)** Heatmap showing Z score scaled the metabolites with higher level at moderate temperature (22°C, 28°C), compared with those at the extreme high temperature (34°C, 40°C). The relative enrichment of each metabolite in different samples were arranged in rows, and the relative levels of all metabolites in each sample were distributed in columns. The grids in heatmap represented metabolites, and shades of color represented relative level of metabolites. Red and blue colors indicate an increase and a decrease of metabolite level, respectively, and white color means no change. Col_22 means seeds germinated at 22°C, Col_28 means seeds germinated at 28°C, Col_34 means seeds germinated at 34°C, Col_40 means seeds germinated at 40°C. **(B)** Heatmap showing Z score scaled the metabolites with the highest level at 28°C, compared with other temperature conditions. The relative enrichment of each metabolite in different samples were arranged in rows, and the relative levels of all metabolites in each sample were distributed in columns. The grids in heatmap represented metabolites, and shades of color represented relative level of metabolites. Red and blue colors indicate an increase and a decrease of metabolite level, respectively, and white color means no change. Col_22 means seeds germinated at 22°C, Col_28 means seeds germinated at 28°C, Col_34 means seeds germinated at 34°C, Col_40 means seeds germinated at 40°C.

As shown in **Figure 2A**, there only 33 metabolites mostly belonging to organic acids/derivates and amines were obviously enriched at 22°C and 28°C, compared to the extreme high temperature, mainly involving in TCA cycle (citrate, succinate, isocitrate), beta-alanine metabolism (spermidine, thiamine, methyltyramine, propynoic acid), phenylalanine metabolism (3-hydroxyphenylacetate, 2-hydroxyphenylacetate), purine metabolism (adenine, adenosine), tyrosine metabolism (4-hydroxycinnamic acid, 3-methoxy-4-hydroxyphenylglycolaldehyde, 3-hydroxyphenylacetate), phenylpropanoid biosynthesis (spermidine, sinapate, 4-hydroxycinnamic acid), carbonhydrates metabolism (D-xylose, acetylglucosamine-1-phosphate) and peptide biosynthesis (kyotorphin). Compared with 22°C, there had 17 metabolites enriched only at 28°C, which were mainly amino acids/derivates including asparagine, valine, tyrosine, glutamine, isoleucine, histidine and threonine, N5-ethyle-L-glutamine and N5-phenyl-L-glutamine, were especially involved in the pathways of cyanoamino acid metabolism (tyrosine, asparagine, isoleucine), glyoxylate and dicarboxylate metabolism (glutamine), ABC transporters (glutamine, histidine) and tyrosine-mediated biosynthesis of various secondary metabolites including phenylpropanoid biosynthesis, glucosinolate biosynthesis (also isoleucine), isoquinoline alkaloid biosynthesis, betalain biosynthesis, ubiquinone and other terpenoid-quinone biosynthesis (**Figure 2B**).

### 3.4 Plant hormones altered during seedling establishment at different temperatures

By using the approach of untargeted metabolomics, some metabolites involving plant hormone biosynthesis were also discovered in this study, especially the biosynthesis of auxin, salicylic acid (SA) and ethylene. All the precursor and intermediates for auxin biosynthesis were detected, which showed that the precursor (tryptophan) and intermediates of the two bypasses including tryptamine, indole-3-acetonitrile, indole-3-pyruvate and indole-3-acetyl-beta-1-D-glucoside to form IAA were enriched at 34°C and 40°C (**Supplementary Figure 1**); whereas, indole-3-acetaldehyde from tryptamine to form IAA was enriched at 22°C and 28°C (**Figure 2A**), indicating that three pathways of IAA biosynthesis responded to different temperature conditions and the bypass from intermediate indole-3-acetaldehyde might be a major pathway for promoting seed germination at moderate temperature. The biosynthesis of indole-3-acetonitrile from glucobrassicin was a unique trait of members of the Brassicales and evidenced to be also used for synthesis of Arabidopsis IAA under drought stress (Hornbacher et al., 2022). In our study, glucobrassicin was higher at 22°C, and indole-3-acetonitrile accumulated at 28°C, which might indicate that this pathway was also used to synthesize the IAA under increased temperature. The intermediates for SA biosynthesis (4-hydroxycinnamic acid, 4-methylsalicylate) was obviously enriched at 22°C and 28°C (**Supplemental Figures S1**; **Figure 2A**), which suggested that SA might be involved in promoting the seed germination at moderate temperature. The intermediates for ethylene biosynthesis, S-methyl-5’-thioadenosine and adenine, were obviously enriched at 22°C and 28°C, and their levels were higher at 22°C than at 28°C (**Figure 2A**), which suggested that 22°C would be optimum for Arabidopsis seed germination, compared with 28°C, although all the seeds were germinated at both temperatures.

### 3.5 Phospholipids and acyl fatty acids altered during seedling establishment at different temperatures

**Figure 3:**
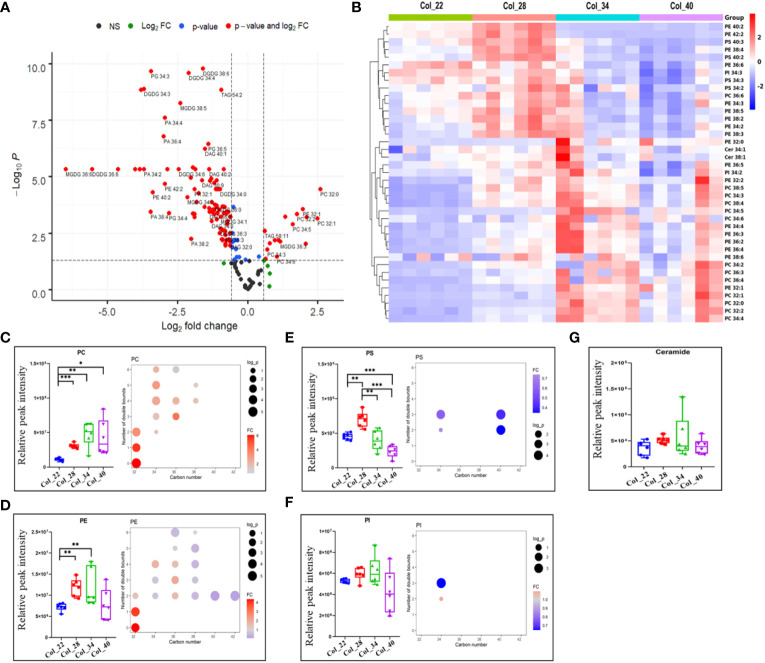
Comparative analysis of lipidome for Arabidopsis seeds germination/seedling growth under four temperature conditions. **(A)** The distinguished alteration of lipids between moderate (22°C, 28°C) and extreme high temperatures (34°C, 40°C) were analyzed by volcano plot. X-axis was Log2 fold change and the y-axis was –Log10 *P* value. The red dots on the left indicated that lipids of seeds germination at moderate temperature significantly reduced level, compared with the extreme high temperature. The red dots on the right indicated lipids level significantly increased. Blue dots represented metabolites with only *P*<0.05 and green dots represent metabolites with only FC>1.5. Gray dots referred to all other identified metabolites with no significant changes between moderate and extreme high temperatures. Phosphatidic acid (PA), Phosphatidylcholine (PC), Phosphatidylethanolamine (PE), Phosphatidylserine (PS), Phosphatidylinositol (PI), Phosphatidylglycerol (PG), Diacylglycerol (DAG), Triacylglycerol (TAG), Sulfoquinovosyldiacylglycerol (SQDG), Monogalactosyldiacylglycerol (MGDG), Digalactosyldiacylglycerol (DGDG). **(B)** Heatmap showing Z score scaled the lipids alteration at moderate (22°C, 28°C) and the extreme high temperatures (34°C, 40°C). The relative enrichment of each lipid in different samples were arranged in rows, and the relative levels of all lipids in each sample were distributed in columns. The grids in heatmap represented lipids, and shades of color represented relative level of lipids. Red and blue colors indicate an increase and a decrease of lipid level, respectively, and white color means no change. Col_22 means seeds germinated at 22 °C, Col_28 means seeds germinated at 28°C, Col_34 means seeds germinated at 34 °C, Col_40 means seeds germinated at 40 °C. **(C–G)** Relative levels of phospholipid classes (small figure on the left of block diagram) and levels of different molecular species (small figure on the right of block diagram) under four temperature conditions. The FC difference was calculated as extreme high temperature group vs. moderate temperature group. Phospholipids shown as PC **(B)**, PE **(C)**, PS **(D)**, PI **(E)** and ceramide **(F)**. Phosphatidylcholine (PC), Phosphatidylethanolamine (PE), Phosphatidylserine (PS), Phosphatidylinositol (PI). Data are presented as mean ± SD. *P < 0.05, **P < 0.01, ***P < 0.001.

Phospholipids function not only as membrane structure but also as messengers to play vital roles for plant development. The volcano plot was used to analyze the significant differences between 22~28°C and 34~40°C, and 104 lipid species were obviously altered among 149 lipid species identified (**Figure 3A**).

**Figure 4:**
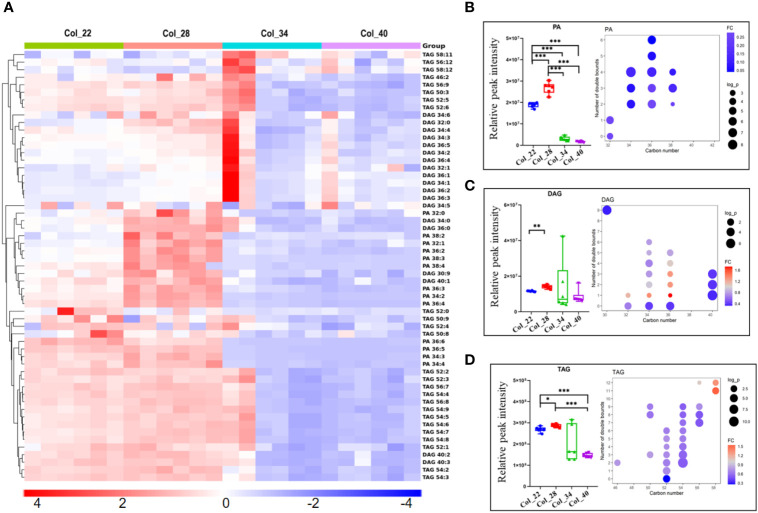
Comparative analysis of phosphatidic acid and glycerol lipids for Arabidopsis seed germination/seedling growth under four temperature conditions. **(A)** Heatmap showing Z score scaled the alteration of phosphatidic acid and glycerol lipids at moderate (22°C, 28°C) and the extreme high temperatures (34°C, 40°C). The relative enrichment of each lipid in different samples were arranged in rows, and the relative levels of all lipids in each sample were distributed in columns. The grids in heatmap represented lipids, and shades of color represented relative level of lipids. Red and blue colors indicate an increase and a decrease of lipid level, respectively, and white color means no change. Col_22 means seeds germinated at 22°C, Col_28 means seeds germinated at 28°C, Col_34 means seeds germinated at 34°C, Col_40 means seeds germinated at 40°C. **(B–D)** Relative classed level of phosphatidic acid and glycerol lipids (small figure on the left of block diagram) and levels of different molecular species (small figure on the right of block diagram) under four temperature conditions. The FC difference was calculated as extreme high temperature group vs. moderate temperature group. Phospholipids are shown as PA **(B)**, DAG **(C)** and TAG **(D)**. Phosphatidic acid (PA), Diacylglycerol (DAG), Triacylglycerol (TAG). Data are presented as mean ± SD. *P < 0.05, **P < 0.01, ***P < 0.001.

**Figure 5:**
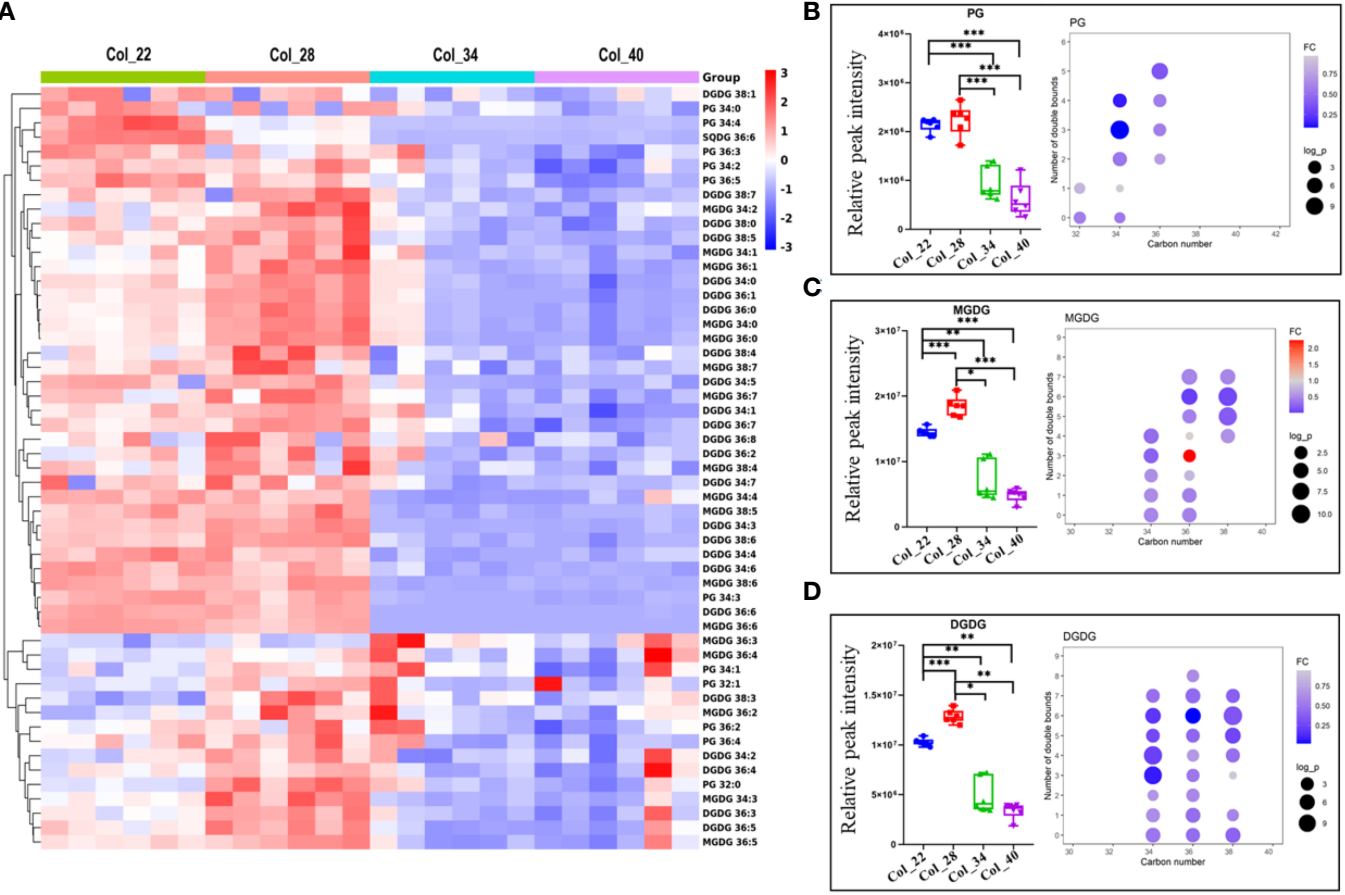
Comparative analysis of phosphatidylglycerol and galactolipids for Arabidopsis seed germination/seedling growth under four temperature conditions. **(A)** Heatmap showing Z score scaled the alteration of phosphatidylglycerol and galactolipids at moderate (22°C, 28°C) and the extreme high temperatures (34°C, 40°C). The relative enrichment of each metabolite in different samples were arranged in rows, and the relative levels of all metabolites in each sample were distributed in columns. The grids in heatmap represented metabolites, and shades of color represented relative level of metabolites. Red and blue colors indicate an increase and a decrease of metabolite level, respectively, and white color means no change. Col_22 means seeds germinated at 22 °C, Col_28 means seeds germinated at 28°C, Col_34 means seeds germinated at 34 °C, Col_40 means seeds germinated at 40 °C. **(B–D)** Lipid classes content differences of phospholipids under four different temperature (small figure on the left of block diagram) and changes in lipid content of different molecular species between low temperature group and high temperature group (small figure on the right of block diagram). The FC difference was calculated as high temperature group vs. low temperature group. Lipids shown here are PG **(B)**, MGDG **(C)** and DGDG **(D)**. Phosphatidylglycerol (PG), Monogalactosyldiacylglycerol (MGDG), Digalactosyldiacylglycerol (DGDG). Data are presented as mean ± SD. *P < 0.05, **P < 0.01, ***P < 0.001.

The hierarchical heatmap analysis Z score scaled the fold changes of phospholipids, which showed that most species of PEs (34:2, 34:3, 36:6, 38:3, 38:4, 38:5, 38:6, 40:2, 42:2) and PGs species (34:0, 34:2, 34:3, 34:4, 36:3, 36:5), and all the species of PSs (34:2, 34:3, 40:2, 40:3) and PAs (32:0, 32:1, 34:2, 34:3, 34:4, 36:2, 36:3, 36:4, 36:5, 36:6, 38:2, 38:3, 38:4) tended to have high levels at moderate temperatures); whereas, other species of PEs and most PCs species tended to have high levels at extreme high temperature (**Figures 3B**, **4A**, **5A**) which indicated that phospholipids were distinguished in response to temperatures. The total lipids classes, PC, PE, PS, PA and PG, showed the significant differences between moderate and extreme high temperature; whereas, PIs and ceramides didn’t exhibit the obvious differences in response to temperatures (**Figures 3C–G**, **4B**, **5B**). PC and PE showed the increased tendency from 22°C and 28°C to 34°C. Oppositely, PS, PA and PG were enriched at 22°C and 28°C. Compared with 22°C, however, both PS and PA were obviously enriched at 28°C. These results suggested the increased tendency of phospholipids in response to temperatures during seed germination and seedling growth. Increased PC during heat stress was suggested to serve as a fatty acyl donors for phospholipid:diacylglycerol acyltransferase 1 (PDAT1) to DAG acylation (Mueller et al., 2017).

The degree of lipid unsaturation was closely related to membrane lipid fluidity, and might affect the physiological efficiency of membrane. The acyl fatty acid (FA) chain length and unsaturation of phospholipids were analyzed in response to moderate and extreme high temperatures, respectively. All the PC species with long FA chain and more unsaturated degree were accumulated at high temperatures; whereas, the high saturated PC species with short FA chain length (PC 32:0 and PC 32:1) tended to be abundant at extreme high temperature (**Figure 3C**). Similarly, the PE species (32:0, 32:1) were also enriched at extreme high temperatures (**Figure 3D**). All the FA chain length and unsaturated degrees of both PS and PI exhibited the similar downward tendency in response to extreme high temperatures (**Figures 3E, F**). All the PA and PG species with long FA chain length and unsaturated degrees were also decreased, especially PA 34:4, 36:4 and PG 34:3, at extreme high temperatures (**Figures 4B**, **5B**). These results suggested that the alteration of FA chain length and unsaturated degrees were closed to their head groups of different phospholipids in response to temperatures during seedling establishment.

### 3.6 Significant differences of glycerolipids and galactolipids during seedling establishment at different temperatures

DAG plays important roles in lipid metabolism, which could generate TAG and were also as substrate to biosynthesize both the phospholipids intermediates PA and galactolipid MDGD. DAG was enriched at 28°C, compared with other temperatures, but DAG did not show significant difference between moderate and extreme high temperatures (**Figures 4A, C**). However, the FA chain of DAG with different unsaturated degrees species showed that DAGs with one double bonds (32:1, 34:1, 36:1) were accumulated at 22°C and 28°C (**Figure 4C**). TAGs were the most abundant in glycerolipids during seedling establishment and were more enriched at extreme high temperatures (**Figure 4D**). The FA chain length and unsaturated degrees analysis showed that the saturated TAGs (52:0) were accumulated at 34~40°C (**Figure 4B**). PDAT1 catalyzed the transfer of a fatty acid from PC to DAG to form TAG and LPC. The seedlings with PDAT1 deficiency had much lower level of TAGs under heat stress, compared to wild-type plants (Mueller et al., 2017). In this study, PC and TAGs accumulation under heat stress (**Figures 3C**, **4D**) might be caused by PDAT1 activation.

PG is essential component of the chloroplast membranes and also as substrate to biosynthesize galactolipids, which showed the similar altered tendency to galactolipids (**Figure 5B**). The alteration of galactolipids (MGDGs, DGDGs, SQDGs) were similar to glycerolipids and enriched at moderate temperatures (**Figure 5A**). The lipid unsaturated degrees of galactolipids were all decreased at extreme high temperatures (**Figures 5C, D**). As previously reported, the decreased galactolipids (MGDG and DGDG) might block plastid biogenesis (Yu et al., 2015).

### 3.7 Metabolites and lipids are critical for seedling establishment at 28°C

**Figure 6:**
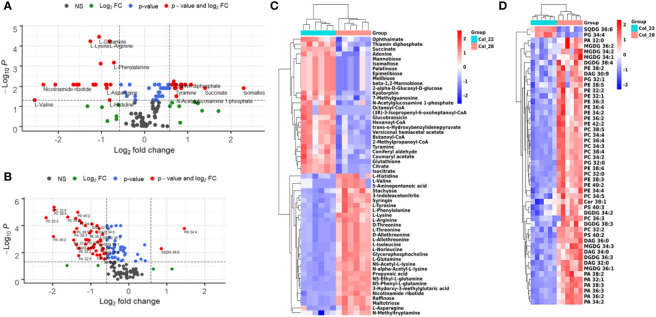
Distinguished analysis of metabolites and lipids for Arabidopsis seedling growth between 22°C and 28°C. **(A)** Distinct metabolites between 22°C and 28°C were analyzed by volcano plot. X-axis was Log_2_ fold change and the y-axis was –Log_10_ *P* value. The red dots on the left indicated that metabolites level of seeds germination at 22°C significantly reduced, compared with 28°C. The red dots on the right indicated metabolites level significantly increased. Blue dots represented metabolites with only *P*<0.05 and green dots represent metabolites with only FC>1.5. Gray dots referred to all other identified metabolites with no significant changes between 22°C and 28°C. **(B)** Distinct lipids between 22°C and 28°C were analyzed by volcano plot. X-axis was Log_2_ fold change and the y-axis was –Log_10_ *P* value. The red dots on the left indicated that lipids of seeds germination at 22°C significantly reduced level, compared with 28°C. The red dots on the right indicated lipids level significantly increased. Blue dots represented metabolites with only *P*<0.05 and green dots represent metabolites with only FC>1.5. Gray dots referred to all other identified metabolites with no significant changes between 22°C and 28°C. **(C)** Heatmap showing Z score scaled the alteration of metabolites between 22°C and 28°C. The relative enrichment of each metabolite in different samples were arranged in rows, and the relative levels of all metabolites in each sample were distributed in columns. The grids in heatmap represented metabolites, and shades of color represented relative level of metabolites. Red and blue colors indicate an increase and a decrease of metabolite level, respectively, and white color means no change. Col_22 means seeds germinated at 22 °C, Col_28 means seeds germinated at 28°C. **(D)** Heatmap showing Z score scaled the alteration of lipids between 22°C and 28°C. The relative enrichment of each lipid in different samples were arranged in rows, and the relative levels of all lipids in each sample were distributed in columns. The grids in heatmap represented lipids, and shades of color represented relative level of lipids. Red and blue colors indicate an increase and a decrease of lipid level, respectively, and white color means no change. Col_22 means seeds germinated at 22 °C, Col_28 means seeds germinated at 28°C.

Based on the distinguished profiles of metabolome and lipidome during seed germination and seedling growth at moderate and extreme high temperatures, we further analyzed the metabolomic and lipidomic differences between the moderate temperatures of 22°C and 28°C. A total of 59 metabolites and 50 lipids were significantly altered between 22°C and 28°C, which included 23 amino acids, 10 sugars/derives, 9 organic acids, 5 CoA, 2 purine/derives, 2 nicotine/derives, 1 indole, 1 phenol, 13 PEs, 10 PCs, 7 PAs, 5 MGDGs, 4 DGDGs, 4 DAGs, 3 PGs, 2 PSs, 1 SQDG and 1 ceramide (**Figures 6A, B**).

The sugars, FAs, nucleotides and polypeptides were obviously enriched at 22°C, which were implicated in carbohydrate metabolism (melibiose, epimelibiose, palatinose, isomaltose, mannobiose, 2-alpha-D-glucosyl-D-glucose), TCA (citrate, isocitrate, succinate, thiamin diphosphate), phosphoenolpyruvate pool (N-acylglucosamine 1-phosphate, 2-methylpropanoyl-CoA), FAs metabolism (Octanoyl-CoA, Hexanoyl-CoA, Butanoyl-CoA), nucleotide metabolism (guanosine, adenine), bioactive cyclin dipeptides kyotorphin (l-tyrosyl-l-arginine) and tripeptides (ophthalmate, glutathione) (**Figure 6C**). However, most of amino acids detected including L-type, D-type and D/L-allothreonine preferred to enrich at 28°C (**Figure 6C**).

Lipids including phospholipids, DAGs, galactolipids showed the different enrichment between 22°C and 28°C. The species of SQDG 36:6 and PG 34:4 were especially low at 28°C (**Figure 6D**). Whereas, other species of lipids including PC (32:0, 34:3, 34:4, 36:4, 38:5), most PE (32:1, 32:2, 34:2, 34:2, 34:4, 36:2, 36:3, 36:4, 38:2, 38:3, 38:4, 38:5, 40:2), PA (32:0, 32:1, 34:2, 36:2, 36:3, 38:2, 38:3), and all the PS were higher at 28°C, compared with 22°C (**Figure 6D**). PG (32:0), DAG (32:0, 36:0), MGDG (34:1, 34:2, 34:3, 36:1, 36:2), DGDG (34:2, 36:3, 38:3, 38:4) with low unsaturated degree also showed high levels at 28°C, compared with 22°C (**Figure 6D**). These results suggested that most of lipids tended to increase to promote seedling growth under moderate higher temperature.

## 4 Discussion

Galactose metabolism plays a vital role in cell wall generation. Galactose was a component of a number of primary polysaccharides, and UDP-D-Gal was a donor nucleotide sugar for the biosynthesis of galactolipids, N-glycans, and various complex carbohydrates in the cell wall (GeorgJ. Seifert et al., 2002). The level of UDP-D-Gal was significantly lower at extreme high temperature of 34°C and 40°C than that at moderate temperature of 22°C and 28°C (**Figures 1D, E**, **2A**). It might handicap cells to proliferate and divide, and then prevented seeds from germination.

During seed germination, energy storage lipid TAGs were hydrolyzed to free FAs and glycerol by TAG lipases to produce energy, which required for the synthesis of sugars, amino acids and carbon chains. The metabolic products from β-oxidation of FAs, and went to TCA cycle for electron transport chain to generate ATP. The glycerol was phosphorylated into dihydroxyacetone phosphate and entered gluconeogenesis. Besides, acetyl-CoA produced by β-oxidation could be sent to glyoxylate cycle and then to gluconeogenesis, which produced the sugars, such as sucrose, to sustain embryonic growth following seeds germination (Quettier and Eastmond, 2009). Sucrose could serve as an osmotic solute and protected the cell molecular structure and membrane stability (Fu et al., 2010; Daloso et al., 2016). Here, we observed that accumulation of sucrose at extreme high temperature, which might indicate that the seed cells suffered from water balance disorder under heat stress.

Amino acids help the synthesis of seed-storage proteins and serve as an excellent source of energy. Hence altering their levels will affect seed germination and seedling growth (Cohen et al., 2016; Xing and Last, 2017). It has been reported that increased lysine content which associated with lower level of TCA intermediates (Angelovici et al., 2009) resulted in lower germination rates by affecting the expression of ribosomal protein genes, stress-associated genes, embryogenesis-associated genes and photosynthesis-associated genes in Arabidopsis seeds (Angelovici et al., 2011). In our study, the level of lysine and methionine was significantly high (**Supplemental Figure S2**) along with low levels of isoleucine and threonine (**Figure 2B**) at extreme high temperature, which may indicate the enhanced conversion from aspartate to lysine. We also found low level of TCA intermediates (citrate, isocitrate, succinate), suggesting that the potential mechanism of failure in seed germination and seedling growth under extreme high temperature may be related to suppress the turnover of embryogenesis-associated genes and the stimulation of expression of photosynthesis-associated genes.

Phospholipids serve as the principle constituent of cellular membranes and play crucial roles in temperature adaption in plants (Harwood, 1991; Harwood, 1994). A high degree of unsaturation increases membrane fluidity and could maintain the physiological efficiency of membrane. Under heat stress, the membrane lipid unsaturation was found to significantly decrease in peanuts, and the levels of saturated and monounsaturated phospholipids were increased in terrestrial plants which were accumulated in the ER and plasma membrane (Higashi and Saito, 2019; Zoong Lwe et al., 2020). In Arabidopsis seeds, the lipid level of PA, PG and PS significantly decreased at extreme high temperature (**Figures 3E**, **4B**, **5B**); whereas, the major membrane lipids, especially PC and PE, showed a completely different trend and their unsaturation degree of PC and PE was decreased (**Figures 3C, D**) at extreme high temperature, which may lead to a less fluidity membrane and then further inhibited seed germination.

Galactolipid formation was initiated by the transfer of a galactose from UDP-galactose into diacylglycerol. These two galactolipids, MGDG and DGDG, represent about 80% of thylakoid lipids in plants (Carter et al., 1956; Bastien et al., 2016). MGDG was synthesized by transferring galactose onto DAG *via* MGDG synthases, and DGDG by galactosylation of MGDG by DGDG synthases (Dörmann et al., 1995). PA, the important precursor for galactoglycerolipids, were essential lipids for membrane formation (Dubots et al., 2012). DAG supplied to MGDG synthases resulted from hydrolysis of PA, and Arabidopsis MGDG synthase (MGD1) was activated by PA and PG (Dubots et al., 2010). PA species with the low unsaturated degree (32:0, 32:1, 34:2, 36:2, 36:3, 38:2, 38:3) and low unsaturated MGDG and DGDG species were enriched at 28°C (**Figure 6D**), indicating that PA, MGDG and DGDG were induced to accumulate by relatively high temperature (28°C). However, the level of PA was dominantly low, and MGDG and DGDG were also in low levels at extreme high temperature (34°C, 40°C) (**Figures 4B**, **5C, D**). Successful germination requires the increase of MGDG and DGDG to allow the formation of plastids in the shoots (Yu et al., 2015; Hu et al., 2018), so the thermoinhibition of seed germination might be caused by blocking the synthesis of MGDG and DGDG, possibly through PA, and then inhibited the seed germination at extreme high temperatures.

The plant hormone, ethylene and its immediate precursor (ACC), was reported to completely improve seed germination (Arc et al., 2013). Ethylene and brassinosteroid affected the balance of ABA/GA through counteracting ABA effects to promote germination (Koornneef et al., 2002). In our study, the biosynthetic intermediates of auxin (tryptamine, indole-3-acetaldehyde), SA (4-hydroxycinnamic acid, 4-methylsalicylate) and ethylene (S-methyl-5’-thioadenosine, adenine) were obviously enriched at 22°C and 28°C (**Figure 2A**), which provided the cues to further explore the functional mechanism of auxin and SA involving in the seed germination and seedling growth. Auxin was reported to be recruited by temperature to modulate higher growth rate of hypocotyl elongation at 29°C, compared with that at 20°C (Stavang et al., 2009). In our study, the higher levels of tryptamine and indole-3-acetaldehyde might also be induced by higher 28°C to biosynthesize IAA for hypocotyl elongation of seedling.

**Figure 7:**
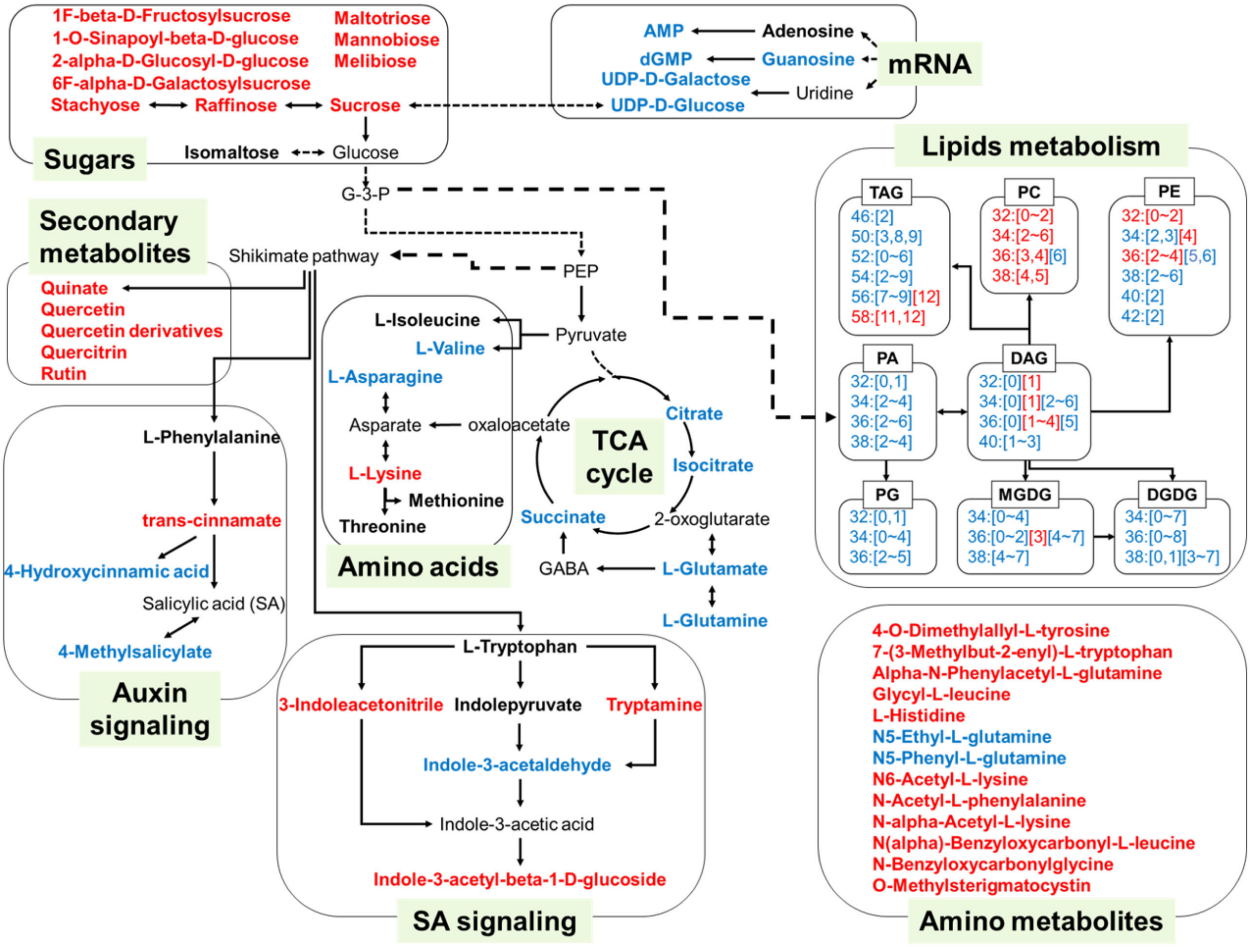
A model illustrating the main alteration of metabolites and lipids during seed germination under heat stress. The metabolites and lipids are implicated in TCA cycle, amino acids metabolism, sugar metabolism, lipids metabolism, secondary metabolites, phytohormone SA and auxin signaling, mRNA transcription during seed germination and seedling growth under heat stress. The metabolites shown in red font indicated significantly increased levels, and shown in blue font indicated the decreased levels under the temperatures of 34°C and 40°C, compared with those under the temperatures of 22°C and 28°C. Lipids were presented as number of carbon atoms and unsaturation degrees. Numbers in bracket were the unsaturation degrees of fatty acyl chains.

Our study demonstrated the metabolic alterations of seed germination under heat stress, including sugars, amino acids, plant hormones and lipids (**Figure 7**), which was cross-validated with some of the previous studies on the transcriptomics of plants under heat stress. Qin et al. found that 22 sugar transporters were responsive to heat stress in wheat leaves and three of them were up-regulated, while 19 of them were down-regulated (Qin et al., 2008). The reduction of sugar transport capacity could cross-validate with sugar enrichment in seeds under heat stress (**Figure 7**). In addition, the increased gene levels in both SA metabolism (Li et al., 2010) and amino acid metabolism (El-Kereamy et al., 2012) under heat stress echoed our results with increased SA and total amino acid content. Heat stress also inhibited gene expression of glycerolipid and prokaryotic glycerolipid metabolism, which cross-validated with decreased content of PG, MGDG and TAG (Higashi et al., 2015). The genes encoding trigalactosyldiacylglycerols (TGDs), which was involved in lipid transfer from ER to chloroplast, together with genes encoding glycerol-3-phosphate acyltransferase (GPATs) and phosphatidic acid phosphatases (PAPs) in the Kennedy pathway, exhibited lower expression levels in heat-stressed seedlings (Zhang et al., 2022). Similarly, the transcriptional levels of these genes were consistent with the reduction of PA and DAG during seedling under heat stress. The reduced activity of the fatty acid desaturase 3 (FAD3A and FAD3B) genes contributed to the decreased degrees of saturation of fatty acids, especially PC and PE under heat stress (Zoong Lwe et al., 2020) (**Figure 7**).

Plant stress memory was reported to make plant become more resistant to future stresses. One possible molecular mechanism of stress memory formation might be chromatin remodeling, which might result in changes in the gene expression pattern that underpins memory response. Another possible mechanism of memory formation involved the changes of several key proteins, signaling metabolites and transcription factors (Sharma et al., 2022). In red seaweed (*Bangia fuscopurpurea*) sublethal exposure to high-temperature stress altered membrane fluidity *via* changes in fatty acid composition, which resulted in the acquisition of heat stress memory and better survival (Kishimoto et al., 2019). The interaction between FORGETTER2 protein phosphatase (FGT2) and phospholipase Da2 (PLDa2) resulted in heat stress memory in Arabidopsis, suggesting that dynamics in membrane phospholipids metabolism and compositional changes underly heat stress memory (Urrea Castellanos et al., 2020). Moreover, sugars not only acted as energy reservoirs during stress periods, but also functioned as signaling molecules. The primary carbohydrate metabolism gene FRUCTOSE-BISPHOSPHATE ALDOLASE 6 (FBA6) together with stem cell regulators CLAVATA1 (CLV1), CLV3, and HEAT SHOCK PROTEIN 17.6A (HSP17.6A) were involved in SAM’s heat stress transcriptional memory (Olas et al., 2021). Phytohormone auxin also played a crucial role in stress memory by stimulating the AUXIN RESPONSE FACTORs expression in the hypocotyl epidermis, which activated cell expansion-promoting genes (Rai et al., 2020). In our study, SA signaling, auxin signaling, amino acids/sugar metabolism, lipid signaling or membrane lipid saturation might be related to acquirement of heat stress memory during seedling development under heat stress.

## 5 Conclusions

Seed germination is a complex physiological process implicated in many metabolic pathways and alteration of metabolites. In this study, we used the untargeted mass spectrometry and advanced ESI to obtain the comprehensive profiles of metabolome and lipidome covering both germinated and non-germinated seedling in response to different temperatures. The results showed that a large of (148 out of total 182) metabolites and specific lipids were highly enriched at extreme high temperatures (34°C, 40°C), which involved in the galactose metabolism, fatty acid degradation, tryptophan/phenylalanine metabolism, and shikimic acid-mediated pathways (alkaloids metabolism and glucosinolate/flavone/flavonol biosynthesis) and phospholipids metabolism (PC, PE). Whereas, TCA, carbohydrates and amino acids metabolism, peptide biosynthesis, phenylpropanoid biosynthesis, IAA biosynthetic pathway and lipids (PS, PA, PG, MGDG, DGDG, SQDG) were featured with high levels at 22°C and 28°C, especially the metabolites of amino acids, trisaccharides, PE, PC, PA, PS, MGDG, DGDG and DAG preferred to enrich at 28°C. In addition, the TCA molecules, disaccharides, nucleotides, polypeptides, biosynthesis of fatty acids, glucobrassicin-mediated IAA and SQDG were obviously high at 22°C. Our results characterized the distinguished metabolites and related regulatory metabolic pathways and the tendency of cellular metabolism pathways in response to different temperature conditions from 22°C to 34°C during seed germination and seedling growth. Our comprehensive metabolome and lipidome especially indicated that 1) phytohormones, such as the bypass of auxin biosynthesis might be activated under different temperatures, and SA might be implicated in the heat stress response; 2) most lipids tended to accumulate under moderate temperatures (22°C and 28°C), whereas the severe heat (34°C and 40°C) contrarily resulted in the decrease of most lipids; 3) most metabolites were induced to be highly enriched for resistance against severe heat stress (34°C and 40°C); 4) organic acids/derivates and amines implicating in cellular TCA, carbohydrates and amino acids metabolism, peptide biosynthesis, phenylpropanoid biosynthesis and IAA biosynthetic pathway were obviously enriched at the moderate temperature (22°C and 28°C). Heat stress is an increasing risk for agricultural crops due to global warming. Our results are crucial to study the comprehensive metabolites and lipids phenotyping, providing the cues to functional innovation with heat tolerance.

## Data availability statement

The original contributions presented in the study are included in the article/**Supplementary Material**. Further inquiries can be directed to the corresponding authors.

## Author contributions

WQ and HY carried out the assays and data analysis of metabolites and lipids. YZ and HY performed the preparation of plants materials. QC and TL extracted peaks and confirmed the structural data. SW and LC preformed the standard rectification of UPLC-MS/MS. HT guided for the data analysis. HY, WQ, YZ and HT wrote the manuscript. All the authors reviewed the manuscript. HY and HT were responsible for contact and communication. All authors contributed to the article and approved the submitted version.
