# Supplementary figures and images for: Comprehensive metabolomic and lipidomic alterations in response to heat stress during seed germination and seedling growth of Arabidopsis
Source: Front Plant Sci. 2023 Mar 29;14:1132881. doi: 10.3389/fpls.2023.1132881 (PMC10090499; doi:10.3389/fpls.2023.1132881)

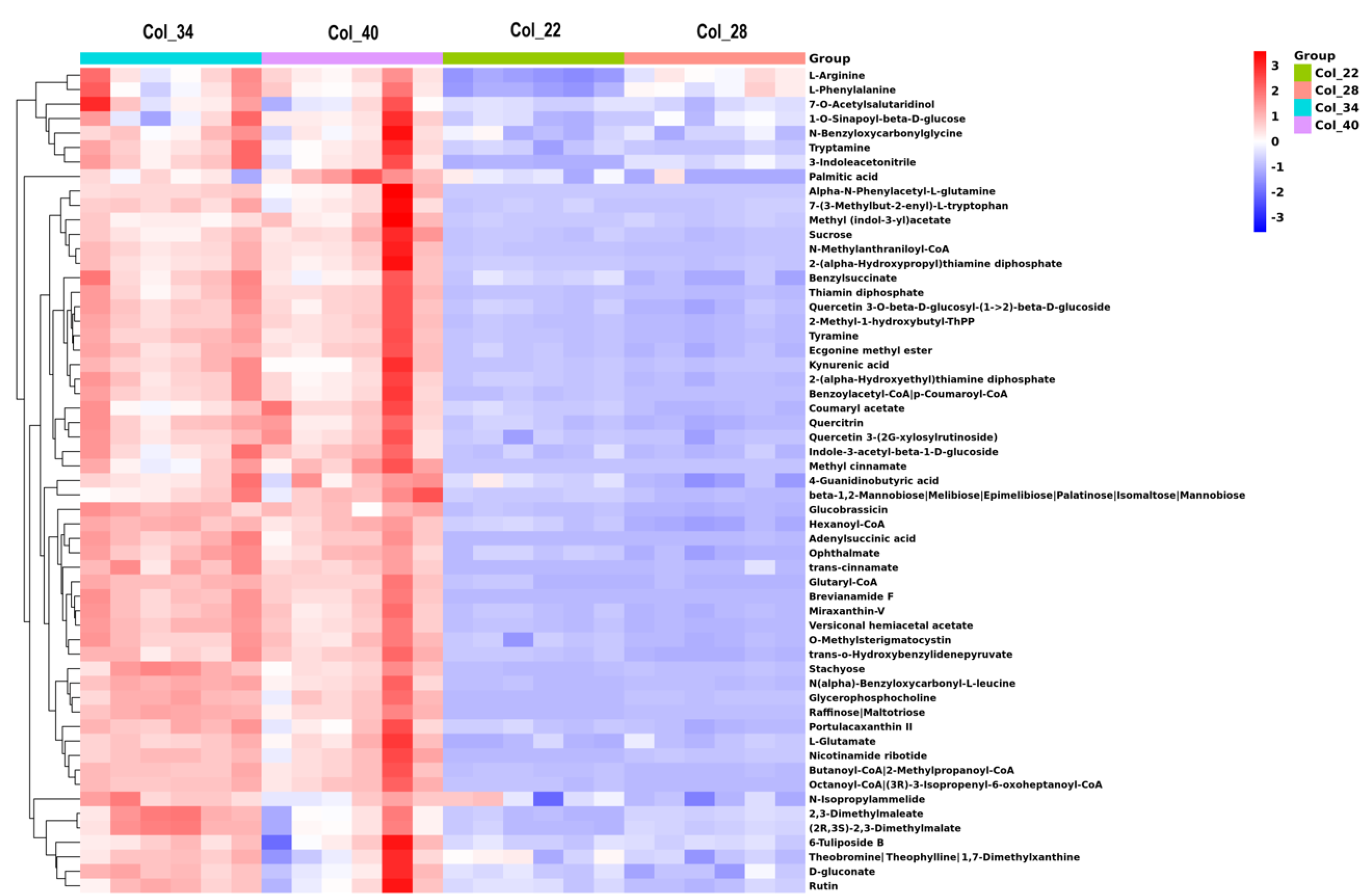

Supplement: Supplementary file 1 [file Image_1.tif]

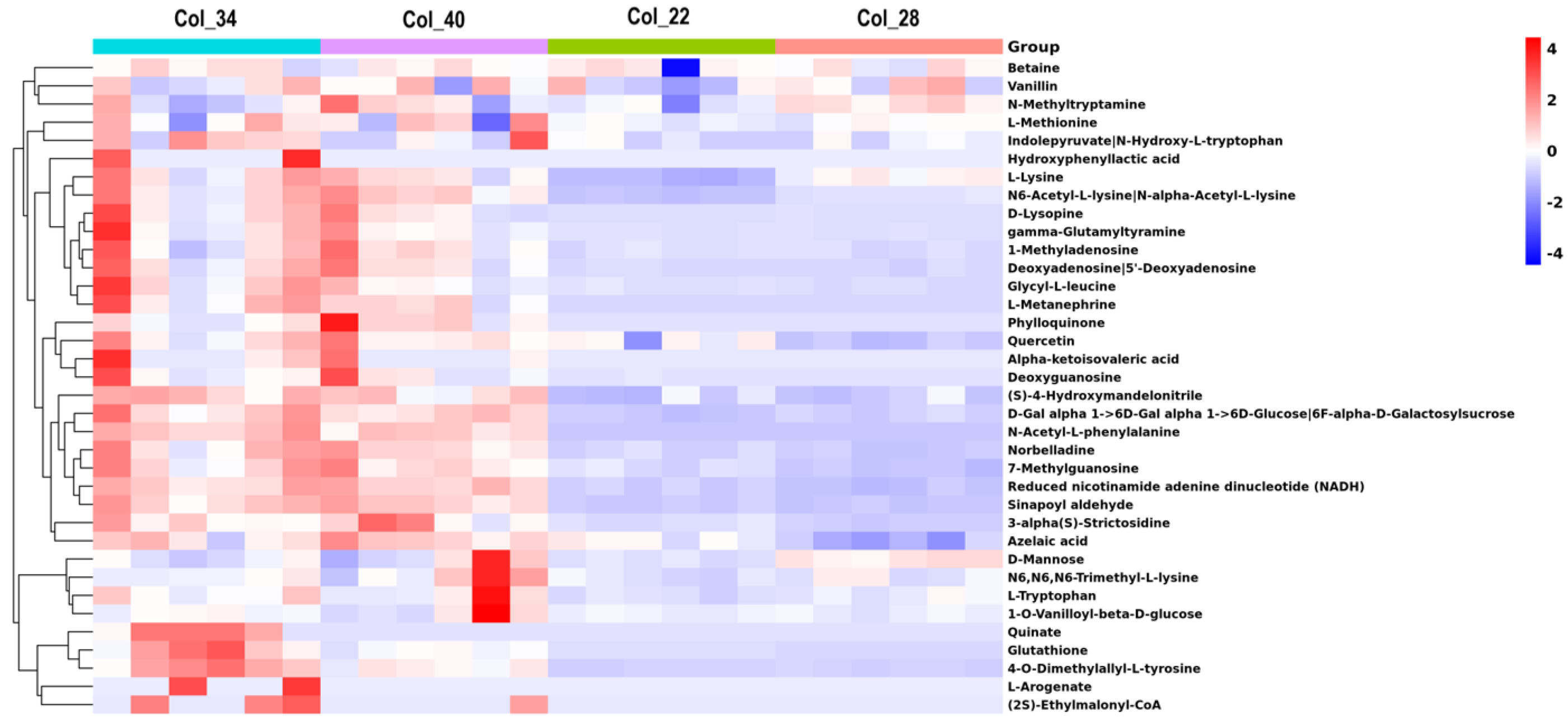

Supplement: Supplementary file 2 [file Image_2.tif]
