# Supplementary material for: Comprehensive metabolomic and lipidomic alterations in response to heat stress during seed germination and seedling growth of Arabidopsis
Source: Front Plant Sci. 2023 Mar 29;14:1132881. doi: 10.3389/fpls.2023.1132881 (PMC10090499; doi:10.3389/fpls.2023.1132881)
Supplement: Supplementary file 3 [file Table_1.docx]

Supplementary Material

Comprehensive metabolomic and lipidomic alterations in response to heat stress during seed germination and seedling growth of Arabidopsis

Wenjuan Qian^1^, Yuxuan Zhu^1^, Qinsheng Chen^1^, Shuaiyao Wang^1^, Longlong Chen^1^, Ting Liu^2^, Huiru Tang^1,^*, Hongyan Yao^1,^*

^1^ State Key Laboratory of Genetic Engineering, School of Life Sciences, Human Phenome Institute, Metabonomics and Systems Biology Laboratory at Shanghai International Centre for Molecular Phenomics, Zhongshan Hospital, Fudan University, Shanghai, China.

^2^ Sciex (China) Co. Ltd, Shanghai, China.

*** Correspondence:**Huiru Tang ([Huiru_tang@fudan.edu.cn](mailto:Huiru_tang@fudan.edu.cn)); Hongyan Yao ([hyao@fudan.edu.cn](mailto:hyao@fudan.edu.cn))

# Supplementary Figures and Tables

## Supplementary Table1 Ionization form and detection parameters in ESI/MS-based shotgun lipidomic analysis

| ID | Pos/Neg | PI/ NL | Mass | Start Mass | Stop Mass | DP | EP | CE | CXP | Comment |
| --- | --- | --- | --- | --- | --- | --- | --- | --- | --- | --- |
| Cer | +H | PI | 246.3 | 450 | 800 | 60 | 10 | 40 | 13 | Based Line 18:1 |
| DGDG | +NH4 | NL | 341.1 | 650 | 1250 | 60 | 10 | 25 | 25 | head Group |
| MAG | +NH4 | NL | 109.2 | 300 | 550 | 60 | 10 | 20 | 13 | head Group |
| MGDG | +NH4 | NL | 179.1 | 600 | 1200 | 60 | 10 | 19 | 25 | head Group |
| PA | +NH4 | NL | 115 | 350 | 950 | 60 | 10 | 25 | 14 | head Group |
| PC | +H | PI | 184 | 350 | 950 | 60 | 10 | 32 | 10 | head Group |
| PE | +H | NL | 141 | 350 | 950 | 60 | 10 | 32 | 16 | head Group |
| PG | +NH4 | NL | 189 | 350 | 950 | 60 | 10 | 30 | 16 | head Group |
| PI | +NH4 | NL | 277 | 350 | 950 | 60 | 10 | 26 | 14 | head Group |
| PS | +H | NL | 185 | 350 | 950 | 60 | 10 | 30 | 13 | head Group |
| SM | +H | PI | 184 | 350 | 950 | 60 | 10 | 38 | 13 | head Group |
| SQDG | -H | PI | 225 | 600 | 1200 | 60 | -10 | -60 | -11 | head Group |
| TAG/DAG | +NH4 | NL | 273.3 | 500 | 1050 | 60 | 10 | 33 | 14 | FA16:0 |
| TAG/DAG | +NH4 | NL | 271.3 | 500 | 1050 | 60 | 10 | 33 | 14 | FA16:1 |
| TAG/DAG | +NH4 | NL | 301.3 | 500 | 1050 | 60 | 10 | 33 | 14 | FA18:0 |
| TAG/DAG | +NH4 | NL | 299.3 | 500 | 1050 | 60 | 10 | 33 | 14 | FA18:1 |
| TAG/DAG | +NH4 | NL | 297.3 | 500 | 1050 | 60 | 10 | 33 | 14 | FA18:2 |
| TAG/DAG | +NH4 | NL | 295.3 | 500 | 1050 | 60 | 10 | 33 | 14 | FA18:3 |
| TAG/DAG | +NH4 | NL | 321.3 | 500 | 1050 | 60 | 10 | 33 | 14 | FA20:4 |
| TAG/DAG | +NH4 | NL | 345.3 | 500 | 1050 | 60 | 10 | 33 | 14 | FA22:6 |
